# Computer-free computational imaging: optical computing for seeing through random media

**DOI:** 10.1038/s41377-022-00725-8

**Published:** 2022-02-14

**Authors:** Yunzhe Li, Lei Tian

**Affiliations:** 1 Department of Electrical and Computer Engineering, Boston University, Boston, MA 02215 USA; 2 Department of Biomedical Engineering, Boston University, Boston, MA 02215 USA

**Keywords:** Imaging and sensing, Adaptive optics

## Abstract

Diffractive Deep Neural Network enables computer-free, all-optical “computational imaging” for seeing through unknown random diffusers at the speed of light.

*Computational imaging*^1^ is an emerging field that seeks to push the fundamental limits in imaging systems by synergistically integrating optics and computation. In recent years, significant advances have been made by leveraging deep learning in computational imaging^2^. By combining novel deep neural network (DNN) architectures and domain knowledge in optical physics, the performance limits in various systems are continuously being re-defined, including spatial resolution^3,4^, depth-of-field^5^, space-bandwidth product^6^, imaging speed^6,7^, sensitivity to low-photon count^8^, and resilience to random scattering^9,10^. Of particular interest by Luo et al.^11^ is the ability to overcome random scattering by a DNN.

A notable advance demonstrated in this work is how the DNN is being designed and implemented to enable “computer-*free*” computational imaging. The majority of the deep learning-based techniques rely on a modern computer (or a computer cluster) to perform both training and deploying the DNNs. As such, the “cost” associated with the deployment (e.g., power consumption, data bandwidth, size, and weight) is fundamentally limited by the computing hardware requirement (e.g., GPUs). In the past few years, *optical computing* solutions, in particular *optical* neural networks (ONNs), emerge as a promising alternative to enable highly efficient “computing” at the speed of light using only optical and photonic components^12^. While optical computing and ONNs have been extensively studied about 30 years ago^13^, including impressive demonstrations, such as face recognition^14^, recent advances in photonic devices and the emergence of extremely large DNNs have re-fueled the research interest in ONNs^12^. Such novel optical computing devices promise to significantly reduce the power, bandwidth, size, and weight and enable “*edge* computing” directly on systems, such as surveillance cameras and autonomous vision systems.

In this work, the diffractive deep neural network (D^2^NN) pioneered by the Ozcan group^15^ is implemented for performing a challenging task for imaging through random diffusers. The D^2^NN is physically constructed by a series of transmissive diffractive surfaces (Fig. 1), in which the “neurons” are embodied by the phase profiles of the “pixels” on the diffractive surfaces and the interconnections are described by the physics of optical diffraction. The D^2^NN is first trained to perform the task of optically reconstructing images of arbitrary objects that are covered by an unknown, random phase diffuser. The result of the training stage consists of a set of phase profiles that are subsequentially fabricated to construct the ONN. A remarkable result demonstrated by the authors is that the trained D^2^NN can all-optically reconstruct the unknown objects through an unknown, new diffuser (that has never been used during training). As such, it implies that this ONN architecture has sufficient generalization power, similar to its digital counterpart^10^, to be robust to random unknown scattering changes. This makes a major advancement in optical computing towards many applications that require robustness against degraded visions, such as astronomical imaging, surveillance, and autonomous driving.

**Fig. 1:**
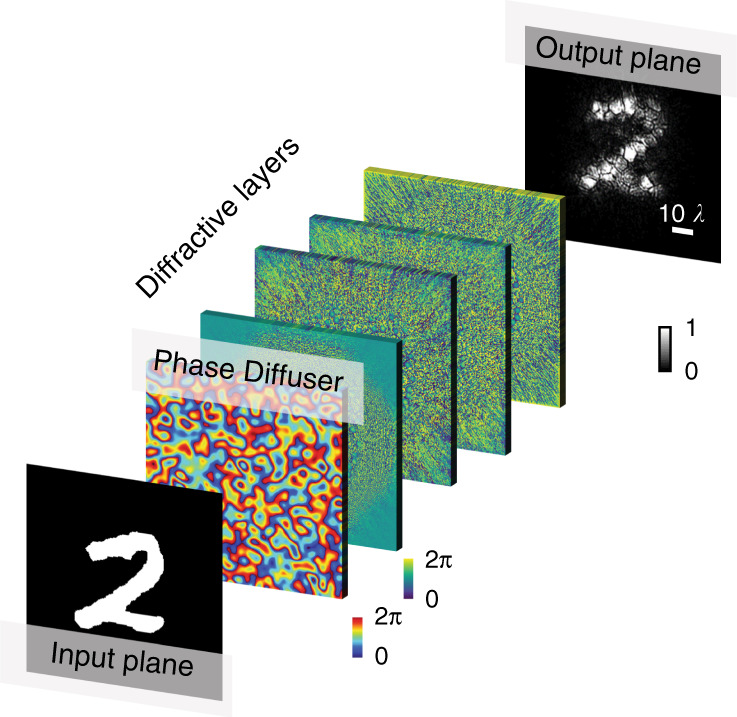
Diffractive deep neural network for computer-free computational imaging through random diffusers. Speed-of-light inference of the letter “2” through the unknown “phase diffuser” using the trained D^2^NN, which consists of multiple diffractive layers.
